# Subdural Empyema Secondary to Severe Paranasal Sinusitis

**DOI:** 10.7759/cureus.31024

**Published:** 2022-11-02

**Authors:** Kruti Ray, Justin Chu, Muhammad I Durrani

**Affiliations:** 1 Emergency Medicine, Inspira Medical Center, Vineland, USA

**Keywords:** sinusitis, subdural empyema, serratia rubidae, recurrent headache, burr hole

## Abstract

A 59-year-old female presented to the emergency department with an acute onset of unilateral facial droop and aphasia. Collateral information obtained from her family revealed a diagnosis of prolonged subacute bacterial sinusitis with initiation of multiple antimicrobial regimens. The patient underwent a non-contrast computed tomography of the brain, which demonstrated a 1.2-cm lobulated extra-axial collection overlying the left frontotemporal region with an associated midline shift consistent with subdural empyema. The patient was initiated on intravenous antibiotics and underwent emergent burr hole placement with successful evacuation of the subdural empyema. Additionally, the patient’s hospital course consisted of a bilateral maxillary antrostomy, bilateral total sphenoethmoidectomy, and bilateral frontal sinusotomy, septoplasty, as and excision of her right concha bullosa. Subdural empyema is a pyogenic infection of the subdural space located between the dura mater and the arachnoid matter, with high rates of morbidity and mortality. Treatment is based on rapid identification and treatment of the underlying causative factor as well as surgical evacuation to obtain source control.

## Introduction

Subdural empyema refers to a loculated collection of purulent material in the subdural space located between the dura mater and the arachnoid mater [1]. In the pre-antibiotic era, subdural empyema was universally fatal, but the introduction of antibiotics has significantly decreased the mortality rate [1]. The most commonly reported etiologies include sinusitis and otogenic infections, meningitis, and neurosurgical procedures, as well as head and neck trauma [2]. When a suppurative process such as sinusitis is implicated, there is a direct invasion into the intracranial compartment and subdural space [2]. Due to the anatomy of the subdural space, a subdural empyema exerts a mass effect, with symptoms arising due to associated inflammation as well as this mass effect on adjacent brain structures [1,2]. The initial diagnosis is often difficult due to non-specific presentation consisting of headaches, fever, vomiting, and generalized malaise that may mimic a variety of conditions. If not rapidly identified, due to the lack of physical barriers in the subdural space, a subdural empyema is able to spread quickly. This often leads to a rapid and fulminant deterioration of the patient [1,2]. Subdural empyema represents a rare but potentially life-threatening entity that necessitates increased awareness and vigilance for clinicians. Although rare, it is paramount to consider this diagnosis as progression of this disease process is associated with neurologically devastating deficits, seizures, meningeal signs, with rapid progression to coma and death. We present a case report of a patient who developed a subdural empyema secondary to subacute paranasal sinusitis with several failed outpatient antimicrobial treatments. This case report serves to add to the body of literature on subdural empyema as well as to reinforce the consideration of subdural empyema if unremitting sinusitis symptoms persist despite multiple antibiotic regimens.

## Case presentation

A 59-year-old female with a medical history of asthma, anxiety, and depression presented to the emergency department with acute onset of confusion, aphasia, and right-sided facial droop. The patient was unable to provide significant history due to her confusion and aphasia. Collateral information obtained from the patient's daughter revealed an onset of symptoms 2 hours prior to arrival to the emergency department. It was noted that the patient had been exhibiting symptoms of intermittent headaches as well as paranasal sinus pressure and intermittent nasal discharge for the preceding month, but review of systems was otherwise negative.

**Figure 1:**
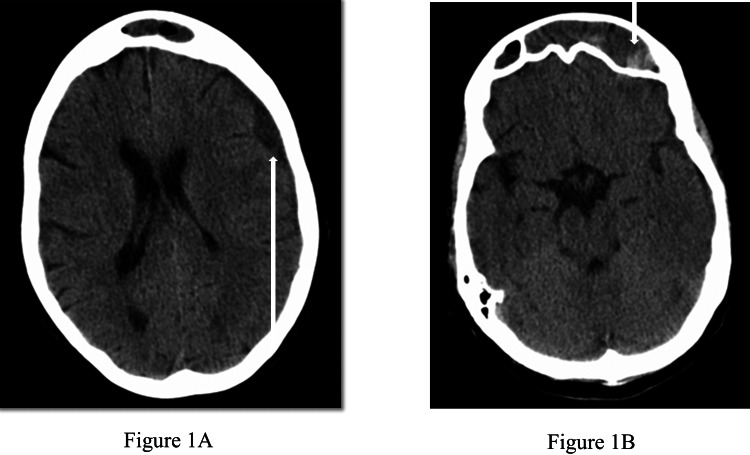
(A) Loculated extra-axial collection overlying the left frontotemporal region with partial effacement of the left cerebral sulci (white arrow). (B) Hypodense region in the inferior portion of the left frontal lobe concerning for abscess (white arrow).

Upon arrival, the patient’s vital signs revealed a blood pressure of 146/81 millimeters of mercury, heart rate of 100 beats per minute, respiratory rate of 16 breaths per minute, temperature of 36.3 degrees Celsius, and 100% oxygen saturation on room air. Her physical examination was significant for minor facial droop with an asymmetric smile and a flat nasolabial fold, severe aphasia, and severe dysarthria. Her physical examination was otherwise normal. Laboratory workup included a complete blood count, complete metabolic profile, and a urinalysis, which were found to be unexceptional. The patient underwent a computed tomography of the brain without contrast, which revealed a loculated 1.2-cm extra-axial collection in the left frontotemporal region consistent with a subdural empyema as well as a hypodense area in the frontal lobe concerning for abscess (Figures 1A, 1B).

**Figure 2:**
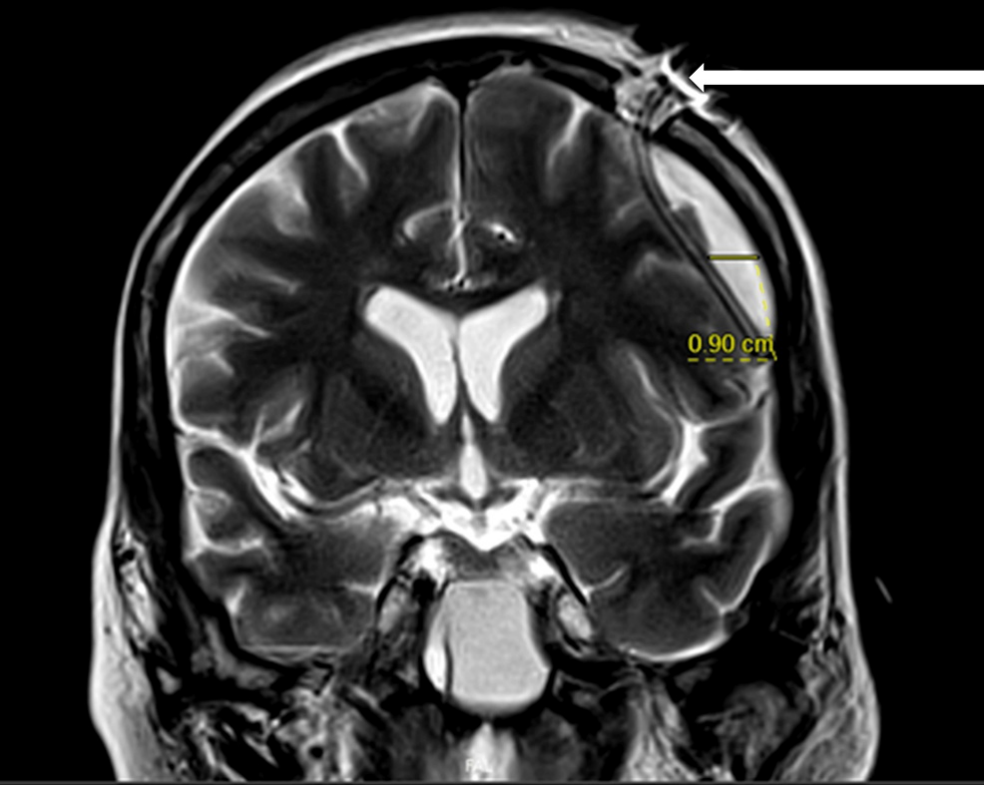
Magnetic resonance imaging status post burr hole procedure with placement of a catheter drain (white arrow).

Given the noted findings, intravenous vancomycin, cefepime, and metronidazole were initiated, and neurosurgical consultation was obtained. The patient underwent emergent burr hole placement with evacuation of the subdural empyema (Figure 2).

**Figure 3:**
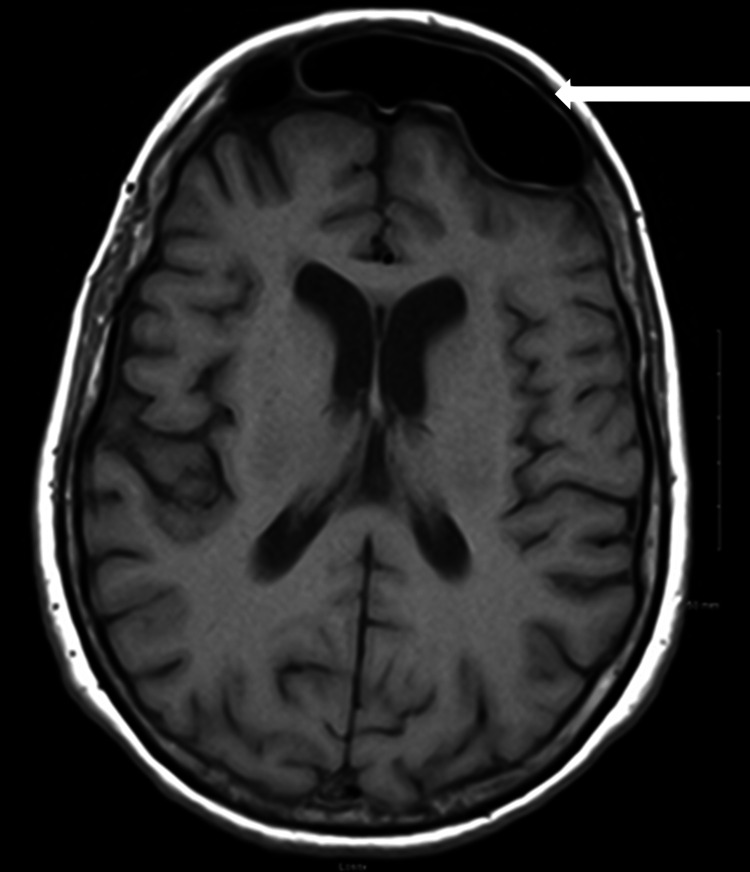
Postoperative axial T1 brain magnetic resonance imaging showing no residual extra-axial collection and post surgical changes of the sinuses with mild mucosal thickening (white arrow).

Additionally, her hospital course consisted of subsequent bilateral maxillary antrostomy, bilateral total sphenoethmoidectomy, bilateral frontal sinusotomy, septoplasty, and excision of the right concha bullosa by otolaryngology. The patient necessitated four weeks of intravenous antibiotic therapy after speciation of bacteria from the culture of her pyogenic intra-operative samples. Post-surgical magnetic resonance imaging studies revealed no residual collections (Figure 3).

## Discussion

The patient had a prolonged post-operative course but was able to recover to her neurological baseline with resolution of her facial palsy, aphasia, and dysarthria. Upon review of the patient’s medical record, it was noted that she had been evaluated at an urgent care center and diagnosed with presumed bacterial sinusitis with initiation of an unknown oral antibiotics. She had finished her antibiotic regimen but continued to exhibit symptoms and was seen by her primary care physician. She was subsequently started on oral clarithromycin for presumed bacterial sinusitis during her follow-up visit. Despite these antimicrobial regimens, she continued to exhibit unremitting symptoms and was evaluated at an outside hospital three days prior to her current emergency department visit for a chief complaint of headache and bilaterally paranasal sinus pressure. No imaging was performed, and she was prescribed amoxicillin-clavulanic acid during this visit and subsequently discharged home with outpatient follow-up. As previously mentioned, in the pre-antibiotic era, subdural empyema was universally fatal, but the use of antibiotics has significantly decreased the mortality rate. As demonstrated here, even with the optimal use of antibiotics, patients may still develop this rare and life-threatening complication of sinusitis. We hope to reinforce this rare complication of sinusitis, which develops in less than 0.1% of patients from sinus or otogeic infections [2]. We also hope to reinforce the consideration of advanced imaging in patients with unremitting symptoms despite the use of antimicrobials. Intracranial infections are life-threatening neurosurgical emergencies that require early recognition and prompt treatment to avoid morbidity and mortality. Prior to widespread adoption of computed tomography imaging, subdural empyema carried a 15-40% mortality rate even with antibiotic treatment [3]. Currently, with advances in technology and widespread computed tomography availability, that mortality rate has gone down to 10% [3]. Subdural empyema accounts for 20% of all intracranial abscesses [1]. They commonly present unilaterally and spread rapidly through the subdural space, leading to vague initial symptoms that give way to a rapid clinical deterioration [1]. Subdural empyema secondary to sinus disease is often due to anerobic and Streptococcus organisms, while gram-negative bacilli and fungi are also implicated in chronic sinusitis [1]. As previously noted, the initial symptoms may be vague and mimic a variety of other conditions. The most commonly encountered early symptoms are headache, followed by fever, altered mental status, vomiting, and seizures [2]. As the infection spreads, focal neurological signs and symptoms develop and may lead to status epilepticus, coma, meningismus, and cranial nerve palsies, as well as aphasia if the dominant hemisphere is involved [1,2]. Laboratory workup is often non-specific for the diagnosis of subdural empyema. Additionally, blood cultures may have poor yield due to the site of the pyogenic infection; thus, intra-operative cultures are often performed when subdural empyema is diagnosed. The initial test for the diagnosis of subdural empyema is often a computed tomography scan of the brain due to its widespread availability. The diagnostic test of choice for subdural empyema is a magnetic resonance imaging study with gadolinium enhancement, which often reveals a fluid collection with a contrast-enhancing rim. Management rests on initiation of intravenous antibiotics along with prompt surgical evacuation to avoid morbidity and mortality.

## Conclusions

Subdural empyema is a rare yet life-threatening condition that should be considered in patients with unremitting signs and symptoms of sinusitis. Advanced imaging with either computed tomography of the brain or magnetic resonance imaging of the brain should be promptly obtained to diagnose such intracranial infections in the right clinical setting. Subdural empyema can have a varied presentation and is associated with significant morbidity and mortality if not promptly recognized and treated. This necessitates increased awareness and vigilance on part of the clinician. This case report should serve to reinforce the clinical presentation and diagnosis, as well as management of subdural empyema. We hope that this case report adds to the body of literature on subdural empyema as well as reinforces the need to obtain advanced imaging in patients presenting with subacute, chronic, or recurrent signs and symptoms of sinusitis.
